# Microlithiasis‐Induced Acute Papillitis Triggers Acute Pancreatitis: Where Do We Go From Here?

**DOI:** 10.1002/ueg2.70211

**Published:** 2026-04-01

**Authors:** Rolf B. Schwarz, Robert C. Verdonk, Marc G. Besselink

**Affiliations:** ^1^ Department of Surgery Amsterdam UMC University of Amsterdam Amsterdam the Netherlands; ^2^ Amsterdam Gastroenterology Endocrinology Metabolism Amsterdam the Netherlands; ^3^ Department of Research & Development St. Antonius Hospital Nieuwegein the Netherlands; ^4^ Department of Gastroenterology St. Antonius Hospital Nieuwegein the Netherlands

Gallstone disease is the leading cause of acute pancreatitis worldwide. Although blockage of the papilla of Vater by gallstones is a well‐known cause of acute pancreatitis, the way in which sludge or microlithiasis causes acute pancreatitis is much more elusive. In this issue of the Journal, Sirtl et al. shed further light on the pathophysiology of microlithiasis induced pancreatitis in their paper “NLRP3 inflammasome activation‐induced acute papillitis as a trigger of acute pancreatitis—a novel mechanism of microlithiasis‐induced acute pancreatitis” [1]. The team of authors is to be congratulated. After previously publishing on the definition of sludge and microlithiasis the present study provides a novel mechanism for microlithiasis‐induced acute pancreatitis [2]. The authors quantified inflammatory infiltrates in human papillary biopsies, assessed NLRP3 activation in mouse macrophages treated with cholesterol and bilirubinate crystals, and used mouse models of microlithiasis‐induced pancreatitis to study gallstone formation, ejection, and its impact on pancreatitis severity. Papillary biopsies of patients (*n* = 4) with microlithiasis‐induced pancreatitis were associated with increased leukocyte infiltration and higher NLRP3 inflammasome activation. In vitro bone marrow‐derived macrophages also showed an increased IL‐1β secretion after stimulation with cholesterol monohydrate and calcium bilirubinate crystals. Microlithiasis formation could be successfully induced in mice due to a high fat diet and devazepide. Mice with caerulein‐induced acute pancreatitis combined with microlithiasis showed higher LDH, GPT and ALP levels, without an increase in pancreatitis severity. In addition, a peri‐papillary infiltrate was found in 2 out 6 mice with microlithiasis. The authors therefore concluded that based on the results a novel mechanism can be proposed in which biliary microlithiasis induce a local inflammatory reaction at the papilla (acute papillitis) via NLRP3 inflammasome activation driven by bone‐marrow derived macrophages, without causing pancreatic outflow obstruction.

So, where do we go from here? This work offers an important conceptual framework, while several key questions remain that invite further reflection and discussion, especially regarding patient treatment.

Clinically, reliable detection of microlithiasis remains challenging. Microlithiasis refers to tiny gallstones or crystals, typically < 5 mm in size, consisting of cholesterol monohydrate or calcium bilirubinate [2]. Although the gallbladder is the primary origin, these can also develop in the common bile duct [3]. Even with endoscopic ultrasound (EUS), currently the most sensitive imaging modality, very small or transient crystals may easily escape detection, either because they are too small to detect or because they have already passed into the duodenum [4]. This is clinically relevant, as undetected microlithiasis will clinically be labeled as idiopathic acute pancreatitis. In addition, endosonographers have limited interobserver agreement when diagnosing biliary sludge [5]. Improving diagnostic strategies will therefore be essential to better identify these patients.

This diagnostic limitation has clear clinical implications for patients with (presumed) idiopathic acute pancreatitis. In the multicenter prospective PICUS trial (Dutch Pancreatitis Study Group), among 105 patients with idiopathic acute pancreatitis, EUS detected an etiology in 32%, most commonly (micro)lithiasis or biliary sludge (23.8%), while the majority remained EUS‐negative [6]. Importantly, recurrence of pancreatitis was higher among patients with negative EUS (17%), and recurrent disease was associated with worse quality of life. These findings are supported by a large retrospective cohort study [7]. This suggests that undetected microlithiasis or transient biliary events may underline a proportion of cases currently classified as idiopathic. Some have suggested that these patients should undergo a cholecystectomy to prevent recurrence of pancreatitis [8]. In their work, Sirtl et all suggest considering cholecystectomy, sphincterotomy or use of ursodeoxycholic acid to recurrent pancreatitis. A randomized trial from Finland indeed reported that laparoscopic cholecystectomy reduced recurrence in patients with presumed idiopathic pancreatitis. However, no EUS was performed in the diagnostic work‐up in this trial [9]. Currently, the ongoing international PICUS‐2 randomized trial is comparing laparoscopic cholecystectomy to conservative management in patients with EUS‐negative idiopathic acute pancreatitis [10]. The results of this study may help determine whether occult biliary disease, such as microlithiasis, plays a clinically meaningful role in these patients and guide future management strategies.

In summary, this well‐designed translational study represents an important and ambitious attempt to clarify the mechanism behind microlithiasis‐induced acute pancreatitis, a clinical entity that remains poorly understood. The study provides a new insight in which microlithiasis does not cause pancreatitis through outflow obstruction, but through NLRP3‐mediated acute papillitis induced by passing biliary crystals; the resulting inflammatory edema may transiently impair pancreatic outflow and trigger acute pancreatitis. Improving our understanding of microlithiasis formation and detection, and therapeutic translation will be essential to move from a proposed mechanism toward improved management of patients with microlithiasis‐associated acute pancreatitis and presumed idiopathic acute pancreatitis.

## Conflicts of Interest

The authors declare no conflicts of interest.

## Data Availability

Data sharing not applicable to this article as no datasets were generated or analyzed during the current study.
